# Microfluidics-Engineered Microcapsules: Advances in Thermal Energy Storage and Regulation

**DOI:** 10.3390/mi16070830

**Published:** 2025-07-20

**Authors:** Yuhan Li, Jian Zhang, Lin Zhuo, Xianjing Wang, Jingyao Sun, Ping Xue, Ke Chen

**Affiliations:** 1 College of Mechanical and Electrical Engineering, Beijing University of Chemical Technology, Beijing 100029, China; li001207rom@163.com (Y.L.); 2024210454@buct.edu.cn (X.W.); xueping@buct.edu.cn (P.X.); 2 Guangxi Construction Testing Center Co., Ltd., Nanning 530000, China; 2023210402@buct.edu.cn (J.Z.); 18689908953@163.com (L.Z.)

**Keywords:** microfluidic, phase-change materials, microcapsules, high throughput, applications

## Abstract

Phase-change microcapsules offer significant advantages for thermal energy storage and regulation. However, conventional mechanical agitation fabrication methods encounter difficulties in achieving monodispersity, precise size control, and structural uniformity. Droplet microfluidics emerges as a promising alternative, enabling controllable production of microcapsules with tunable sizes (1–1000 μm), programmable core–shell configurations, and high encapsulation efficiency. This review comprehensively summarizes recent advances in microfluidic strategies for phase-change microcapsules fabricating, including single encapsulation, multi-core encapsulation, and high-throughput parallelization and their applications in solar energy storage, building thermal regulation, electronics cooling, and smart textiles. The review highlights key challenges for future advancement which will unlock the full potential of microfluidics-engineered phase-change microcapsules in next-generation thermal energy technologies.

## 1. Introduction

**Figure 1:**
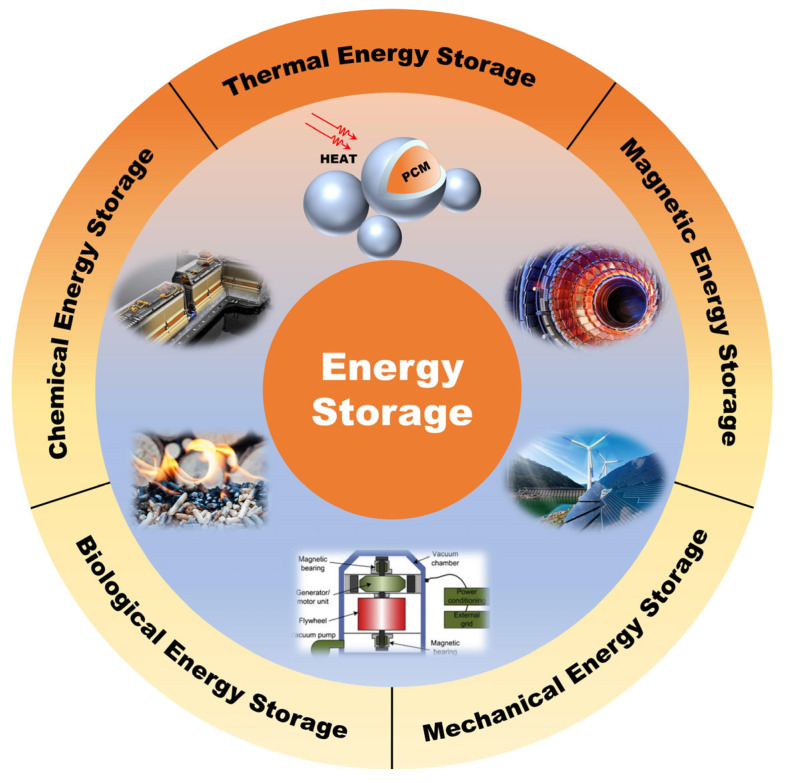
Schematic overview of energy storage technology.

Since the Industrial Revolution, the concentration of carbon dioxide in the atmosphere has persistently increased, currently reaching a level of 419 ppm [1]. At the 28th Conference of the Parties to the United Nations Framework Convention on Climate Change (COP28) in 2023, governments of various countries have reached a consensus on the necessity of phasing out fossil fuels in the energy system [2]. Greenhouse gas pollution and energy shortages have emerged as critical drivers of energy system conversion and upgrading. Energy storage, as a critical technology for energy conversion, is increasingly attracting the attention of numerous researchers for its ability to effectively collect and transfer clean energy. According to storage media, energy storage technology mainly includes thermal energy storage (TES), chemical energy storage, mechanical energy storage, biological energy storage, and magnetic energy storage, as shown in Figure 1 [3,4]. Among them, TES has unparalleled advantages in terms of its exceptional flexibility, such as the flexibility of cold and hot transportation and the integration of volatile energy supply [5].

TES is a technology which captures and stores thermal energy in media for later application. It can efficiently capture and store heat from primary sources, such as solar thermal energy, geothermal energy fossil-fuel power, nuclear power, industrial waste heat and biomass [6]. TES includes sensible heat storage (SHS), latent heat storage (LHS). Attention from academia and industry has been focused on the application of energy storage materials in LHS. Storage media for LHS, which are also called phase-change materials (PCMs), should have characteristics such as high specific heat capacity, long-term thermal stability, and low cost [7,8]. PCMs complete the energy storage process almost exclusively at a constant temperature, making it easier to control the process of heat storage and release. Better thermal stability and density of PCM lead to a wide range of applications in fields, such as buildings [9,10], medical caring [11,12], textiles [13], thermal protection [14], renewable energy [15], and solar heating [16]. Several typical phase-change materials are summarized in Table 1.

Furthermore, polymer-based phase-change materials constitute another significant research focus. Wu et al. presents a novel strategy for fabricating recyclable solid–solid phase-change materials via reversible anhydride-alcohol crosslinking. Using maleic anhydride alternating copolymers and polyethylene glycol as crosslinker/phase-change component. The resulting PCMs attain a peak latent heat value of 156.8 J g^−1^ and exhibit certain flexibility and a tunable tensile strength ranging from 6.6 to 11.0 MPa [21]. Geng et al. develops a biodegradable polylactic acid/polyethylene glycol (PLA/PEG) phase-change composite via melt blending at 180 °C, achieving a record 90 wt% PEG loading where PLA forms a 3D network structure. The material exhibits high latent heat of 163.7 J/g, excellent thermal cyclability (>95% enthalpy retention after 100 cycles), and processability, with leakage tests confirming shape retention at 80 °C for 30 min even at 90 wt% PEG [22]. Novel polymer phase-change materials are advancing toward breakthroughs in multifunctional integration, environmental friendliness, and intelligentization, while novel forming processes for these new materials also require significant breakthroughs.

## 2. Phase-Change Microcapsules by Droplet Microfluidic

Phase-change materials exhibit excellent heat storage and photothermal properties. However, during the manufacturing, design, and utilization of PCM, problems such as leakage, corrosion, and volume changes may occur [23]. The packaging form and structural design of PCMs have become a focal point for addressing these challenges.

**Figure 2:**
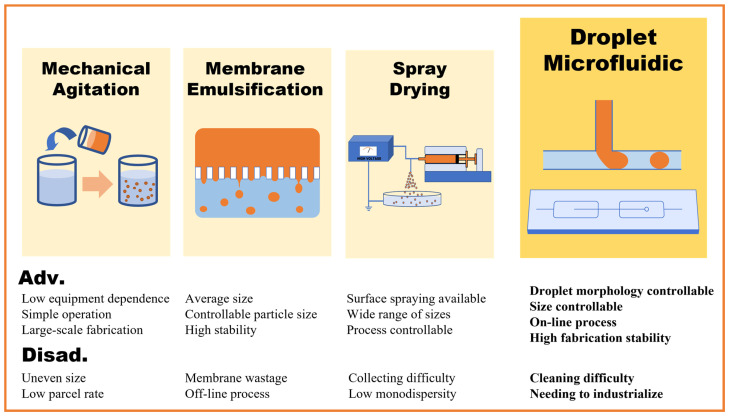
Advantages and disadvantages of common phase-change microcapsules fabrication techniques.

Phase-change microcapsules are functional particles with a core–shell structure that provide protection for phase-change materials and good isolation from external pollutants. The reduction in geometric scale brings about an increase in surface area, thereby promoting the improvement of heat transfer and energy storage efficiency. Recently, researchers have developed various technical routes for fabricating phase-change microcapsules. Their advantages and disadvantages are summarized in Figure 2. The mechanical agitation method heavily relies on fluid shear-based mechanical stirring, which makes it difficult to control the formation of microcapsules and the uniformity of the formed microspheres [24]. Droplet microfluidic is a versatile method for preparing single and double emulsion droplets, microspheres, and microcapsules. It offers advantages over other emulsification methods, including controllable droplet shapes and categories, ease of integration for high-throughput production, and adjustable droplet sizes within a certain range [25,26,27].

Droplet microfluidics, capable of generating single and multiple emulsion droplets, microspheres, microcapsules, etc., exhibits broad applicability relative to other emulsified microsphere fabrication methods. It enables controllable droplet morphologies and moldings, facilitates integration for high-throughput production, and affords tunable droplet sizes within a given range. This technology holds potential across diverse applications medical detection [28], polymer materials [29], etc.

**Figure 3:**
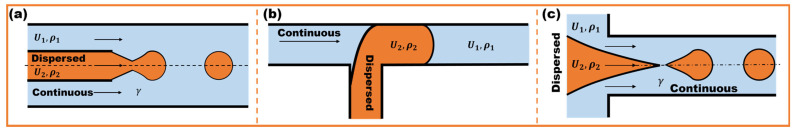
Geometries of three common methods of droplet microfluidics; (**a**) co-axial flow method; (**b**) T-junction method; (**c**) flow-focusing method.

Droplet microfluidics is a production technology for microspheres and microcapsules. It has a wide range of applications in various microfluidics-related fields due to its excellent controllability. Microfluidics was first proposed by Manz et al. [30] in the 1990s and has since been continuously developed and extended. It is currently used in various fields, including mechanical engineering, biomedical engineering, life sciences, food engineering, and materials science. In the last decade, microfluidics has made significant contributions to bioengineering, such as its use in drug delivery [31] and cell biology [32]. The essence of droplet microfluidics is the shearing of a dispersed phase fluid into droplet microspheres within microscopic flow channels, driven by multiphase fluid shear and surface tension [33]. Currently there are three main common microfluidic embedding methods: the co-axial flow method, the T-junction method, and the flow-focusing method, as shown in Figure 3.

Cramer et al. proposed the co-axial flow method, which utilizes a concentric arrangement of capillary tubes to create droplets within an annular fluid stream, as shown in Figure 3a [34]. The method involves two capillary tubes: the inner capillary tube contains the dispersed phase, and the outer capillary tube contains the continuous phase. The dispersed phase flows from the inner capillary tube into the continuous phase, where it is constantly subjected to the continuous phase. During the process of flowing from the internal capillary to the continuous phase, the internal dispersed phase is continuously sheared by the continuous phase. Thorsen et al. proposed the T-junction method. Two microfluidic channels are perpendicular to each other, as shown in Figure 3b [35]. At the intersection, the immiscible fluids undergo shear, with the continuous phase shearing the dispersed phase. When the interfacial tension reaches a critical point, the dispersed phase breaks up to form a microdroplet at the intersection [36]. Anna et al. proposed the flow-focusing method, which is dominated by capillary instability, as shown in Figure 3c [37]. The continuous phase flows in from both sides of the dispersed phase, producing an entrainment focusing effect on the dispersed phase, which causes the dispersed phase to rupture and form droplets [38].

The microfluidic chip, as the main device of microfluidics fabrication, is a microfluidic flow channel on a centimeter scale. The chip can carry out reactions, shear, fluid flow, and other functions, embodying the concept of ‘lab on a chip’ [39]. The fabrication of microfluidic devices is crucial for ensuring the consistency of droplet formation and the accuracy of droplet control. Common materials used for microfluidic chips include glass, polymethyl-methacrylate (PMMA), and polydimethylsiloxane (PDMS), etc. Usually, soft lithography is used for the processing of organic polymer chips [40]. The design of microfluidic chips used for embedding microspheres and microdroplet emulsions should consider the interaction between the chip material and the different phases involved in the process.

### 2.1. Monodisperse and Core–Shell Microspheres

**Figure 4:**
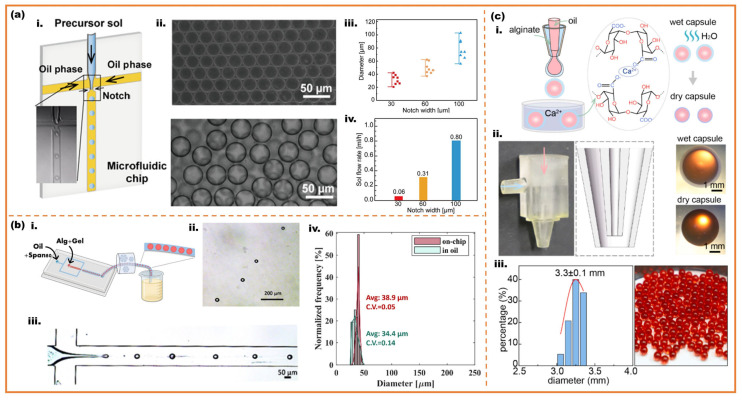
Fabrication of monodisperse microspheres through microfluidics; (**a**) microfluidic-assisted sol–gel monodisperse mesoporous silica microspheres: (**i**) schematic diagram of microfluidic-assisted droplet preparation; (**ii**) optical photograph of the monodisperse droplets; (**iii**) the size range of monodisperse droplets prepared with different notch widths; (**iv**) influence of the notch width on the silica sol flow rate [44]; (**b**) thermo-controlled microfluidic generation of monodisperse alginate microspheres: (**i**) schematic illustration of the process; (**ii**) optical photograph of microspheres; (**iii**) generation of monodispersed droplets at the cross-junction; (**iv**) size distribution of Alg-RGD-Gel droplets at generation and Alg-RGD-Gel monodisperse microspheres in oil with CaCl2 nanoparticles [45]; (**c**) preparation of monodisperse biocompatible core–shell microcapsules: (**i**) schematics showing the preparation of core–shell capsules; (**ii**) microfluidic devices and optical photograph of the capsules; (**iii**) size distribution of dry capsules [46].

Monodisperse microspheres of phase-change materials are fabricated with only two phases, including water-in-oil (W/O) and oil-in-water (O/W) [41,42]. These microcapsules typically exhibit a core–shell architecture, where PCM constitutes the core material enveloped by a polymeric shell [43]. Zhang et al. presented a microfluidic-assisted sol–gel strategy for precise control of monodisperse mesoporous silica microspheres using flow-focusing, as shown in Figure 4a [44]. By regulating flow rates and channel sizes, they achieved microspheres diameter (20–100 μm) offering a versatile platform for fabricating functional silica microspheres. Chen et al. presented monodisperse modified alginate microspheres through flow-focusing droplet microfluidics and a thermo-controlled microfluidic strategy for off-chip external gelation. Successful production of highly spherical microspheres with a diameter of 27–46 μm (CV = 0.14) is shown in Figure 4b [45]. Zheng et al. illustrated the fabrication process and critical performance characterization of alginate-based core–shell capsules through microfluidics in the accompanying Figure 4c. The study proposed monodisperse biocompatible core–shell capsules with a diameter ranging from 3 to 3.5 mm through co-axial flow microfluidics [46].

Monodisperse microspheres and microcapsules can be stably fabricated within the size range of micrometers to millimeters using a microfluidic-controlled approach. However, the preparation of microcapsules typically necessitates the formation of a three-dimensional network structure through the interaction between calciumions (Ca^2+^) and carboxyl groups (-COO-) on alginate chains, or alternatively, the incorporation of additional forces such as gravity or electromagnetic effects to facilitate core–shell formation. The encapsulation of phase-change materials in a core–shell structure imposes relatively stringent requirements on the shell.

**Figure 5:**
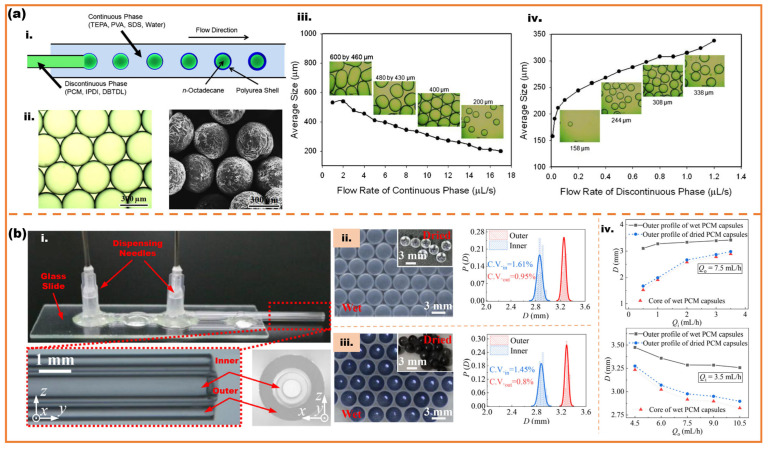
Generation of phase-change microcapsules with droplet microfluidic; (**a**) N-octadecane with polyurea shell fabricated by co-axial droplet microfluidic: (**i**) schematic diagram of the fabrication of monodisperse microcapsules; (**ii**) optical photograph of microcapsules; (**iii**) effects of the flow rate of continuous phase on the size of the microcapsules; (**iv**) effects of the flow rate of discontinuous phase on the size of the microcapsules [48]; (**b**) device and generation of RT25 with multi-layer graphene microspheres: (**i**) gravity-assisted co-flowing microfluidic device; (**ii**) optical photograph of wet PCM capsules without multilayer graphene and corresponding CV values for size; (**iii**) optical photograph of wet PCM capsules with 2 wt% multilayer graphene and corresponding CV values for size; (**iv**) diameters of the PCM capsules versus the rates of the inner flow Q_i_ and outer flow Q_o_ [49].

The phase-change microcapsules’ shells demonstrate adequate mechanical robustness and thermal stability to ensure reliable performance across varied environmental conditions [47]. Lone et al. approached a method to fabricate phase-change microcapsules in the size of 35–500 μm. N-octadecane, the phase-change material, is contained in the polyurea by the co-axis droplet microfluidic method, as shown in Figure 5a. The sizes and encapsulation rates can be controlled by adjusting the flow rates of continuous and dispersed phases [48]. Hao et al. have implemented the incorporation of multi-layer graphene into PCM (RT25) utilizing a co-axis droplet microfluidic method to enhance the thermal conductivity of PCM capsules, as shown in Figure 5b. The team conducted experiments to illustrate that the reliability and size of the core–shell structure of PCM capsules can be precisely controlled by adjusting the flow rates of the inner and outer phases via droplet microfluidics, thereby achieving highly monodisperse thermo-regulation microcapsules (CV ≤ 2%) [49].

Due to the constraint imposed by single embedding, the continuous phase typically serves as the shell in droplet microfluidic devices, where only the embedding process occurs. Consequently, alternative methods are often employed to aid in the formation and curing of their microcapsules, including gravity and magnetic assistance [50,51]. To achieve additional functionalities and corresponding structures, the double-embedding one-step forming method is commonly employed in the microfluidic production of microencapsulated droplets of PCM.

The microspheres fabricated by multiple emulsion are designed to be suitable for containing larger droplets or multiple inner compartments due to their structural peculiarities [52]. The multiple emulsion microspheres can be classified into multiple cored, Janus, and multiple compartment [53,54,55]. Two prevalent configurations of multiple-core embedding systems are involved: multi-layered nesting and paratactic cores within a single shell. The former entails a multi-layered structure achieved through iterative embedding processes, while the latter involves parallel embedding of cores within the outermost phase during the molding process. The structures of double emulsion microcapsules typically include W/O/W and O/W/O as the normal multiple cored microspheres [56]. The fabrication of multiple emulsion microspheres mostly arises from the amalgamation of three fundamental microfluidic methods, namely the double T-junction method [57], co-axial convection method [58], and double flow-focusing method [59].

**Figure 6:**
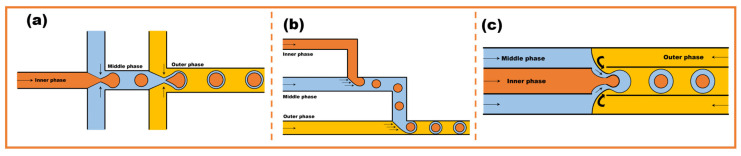
Geometries of multiple sheared microcapsules; (**a**) multiple flow-focusing method; (**b**) multiple T-junction method; (**c**) multiple co-axial convection method.

Flow-focusing microfluidic is usually classified into one-step and multi-step forming method, as shown in Figure 6a [60,61]. Azarmanesh et al. briefly discussed the potential transition between the two-step and one-step formation regimes, as well as the fluctuations in shell thickness [62]. The planar flow-focusing cross-junctions are commonly preferred due to their capability to integrate with other devices and ensure precise size control [63].

Okushima et al. introduced the two-step T-junction method for the preparation of W/O/W double emulsions, as shown in Figure 6b [64]. Initially, the water and oil phases are first emulsified at the primary junction, resulting in the formation of W/O droplets. They undergo a secondary emulsification step at the subsequent junction to form W/O/W droplets. Hydrophobic treatment is conducted at the primary junction, while hydrophilic treatment is applied at the secondary junction. Due to its simple structure, T-junction microfluidics can be readily arranged in parallel or in series, facilitating high-throughput or multiple embedding applications [65]. Nevertheless, owing to the fundamental principles governing the interplay between viscosity and surface tension, the viscosity ratio of the fluid plays a significant role in droplet formation [66], rendering the control of droplet size inherently complex.

Co-axial convection refers to a flow-focusing microfluidic technique wherein two capillaries are nested coaxially within an axial channel, as shown in Figure 6c [67]. Wang et al. implemented W/O/W highly monodisperse microcapsules using the axial convection microfluidic method [68]. The research team utilized UV curable material to cover the phase-change materials.

Due to its unique one-step molding advantages, multiple embedded microfluidics also exhibit significant potential in PCM microsphere embedding, particularly for phase-change microcapsules with specific structural demands. Akamatsu et al. utilized glass capillary devices to encapsulate n-tetradecane and n-hexadecane within silicone-based shells, which were subsequently solidified through UV irradiation [69]. Han et al. employed the co-axial convection, the nested capillary microfluidic device, to encapsulate n-Hexadecane with polyethylene glycol diacrylate and silicone oil as middle-phase and outer-phase, which achieves a high thermal performance and heat charging rate [70].

Special-structured microspheres (such as Janus microspheres) can also be prepared by microfluidic methods. Nisisako et al. presented an innovative microfluidic technique for fabricating oil-filled polymeric microcapsules with independently tunable size and shell thickness, leveraging the structural evolution from Janus droplets to core–shell configurations. Utilizing a T-shaped cross-flow droplet generator on a glass chip, biphasic Janus droplets are produced in a single step which transition to monodisperse core–shell structures via interfacial energy minimization, primarily driven by polyvinyl alcohol adsorption, resulting in a silicone oil core encapsulated by a photocurable acrylate shell; the size is precisely controlled within 70–150 μm by adjusting the aqueous flow rate (Qw), with coefficients of variation as low as 1.3–3.3% [71].

### 2.2. High-Throughput Microfluidic

Microfluidic technology has been well known for its ability to efficiently manage laminar flow, facilitate continuous operations, precisely fabricate droplets, and provide excellent product performance [72,73]. However, the production rate of traditional microfluidic droplet generators ranges from tens to hundreds of microliters per minute, which is evidently insufficient for meeting the industrial application standard [74]. Scaling-up strategies have been utilized to address the problem. The primary trend in high-throughput microfluidics involves the integration of individual droplet microfluidic units into a single cohesive device [75]. The common units of microfluidic droplet generation are the co-axial unit [76], T-junction unit [77], flow-focusing unit [78], and step emulsification unit [79].

Recently, the realization of microfluidic high throughput is generally based on the parallel amplification of multiple microfluidic units, and the microfluidic unit is integrated into a fluid distribution network. Each phase of fluid within this network necessitates only a single pump for fluid transportation, obviating the need for individual pumps for each microfluidic chip unit. This approach significantly reduces capital investment [80,81,82]. Hashimoto et al. employed a flow-focusing unit array configuration for the generation of hexadecane droplets [83]. The formation of liquid droplets was found to remain independent, contrasting with bubble formation, which depends on interactions between adjacent generators. This offers a theoretical possibility for achieving high-throughput fabrication of phase-change microcapsules using the flow-focusing method.

The common methods of parallel amplification high-throughput technology are classified into two-dimensional matrix arrangement and branches arrangement [84]. Furthermore, step emulsification is also widely employed in scaling up microfluidics [85,86]. The formation of droplets arise from the interaction between interfacial tension and the Laplace pressure difference [87,88].

**Figure 7:**
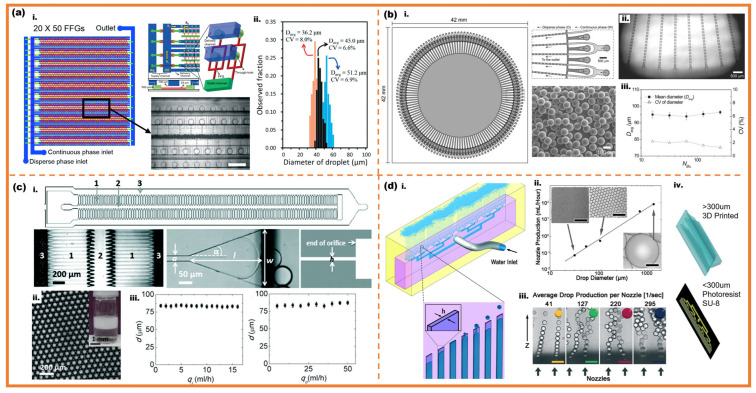
Scaled-up droplet generation with microfluidic; (**a**) kilo-scale droplet high-throughput generation [91]: (**i**) schematic diagram and optical images of kilo-scale droplet generation; (**ii**) size distribution for generated droplets with different ratio of the flow rates; (**b**) flow-focusing units circularly allocated [92]: (**i**) schematic of 128 cross-junctions on a chip and optical images of microspheres; (**ii**) optical images of formation of 128 cross-junctions; (**iii**) size distribution of the droplets produced with different numbers of junctions; (**c**) monodisperse droplets generation with step emulsification units [93]: (**i**) schematic illustration of the millipede device; (**ii**). optical images of microspheres; (**iii**) the diameter of droplets produced with different flow rates; (**d**) ‘volcanic device’ with 400 step emulsification units arranged in parallel [94]: (**i**) schematics of ‘volcanic device’; (**ii**) maximum achievable flow rates through individual nozzles dependent on droplet diameter; (**iii**) optical images of produced droplets at different flow rates, (**iv**) moldings for volcano devices.

In order to achieve high-throughput fabrication with microfluidics, it is essential to design microchannels and units reasonably so that the flow rate allocated to each unit is basically the same [89,90]. Jeong et al. developed a 3D kilo-scale droplet high-throughput generation with 1000 parallel flow-focusing units into one piece, as shown in Figure 7a [91]. The device measured 6 × 5 cm^2^ and comprised 1000 flow-focusing units, arranged in 20 rows with each row containing 50 units. The generation achieved a throughput of 1.5 L/h with 45 μm diameter W/O emulsion droplets, and the CV value was 6.6%. Nisisako et al. arranged flow-focusing units circularly on a 4 × 4 cm^2^ chip which achieved a throughput of 320 mL/h and a mean diameter of 96.4 μm with a CV of 1.3%, as shown in Figure 7b [92]. The method was also proved to produce Janus droplets at a throughput of 120 mL/h and a CV of 3.3%. Amstad et al. proposed a 20–200 μm monodisperse droplet generation with step emulsification units, as shown in Figure 7c [93]. The parallelized microfluidic device, which was called ‘the millipede device’, arranged 550 individual step emulsification units. At the end of each unit gathered in an outlet which led to a throughput of 700 mL/h with a CV of 5%. Stolovicki et al. also utilized 400 step emulsification units arranged in parallel, as shown in Figure 7d [94]. The dispersed phase flows in from the bottom of the device and upward due to the buoyancy of the liquid, therefore the device was described as a ‘volcanic device’. Through the process of using continuous phase shearing, the dispersed phase can produce droplets in the range of 30 μm to 1000 μm, which can be produced per hour. The 10 L dispersed phase achieves the high-throughput preparation of droplets.

**Figure 8:**
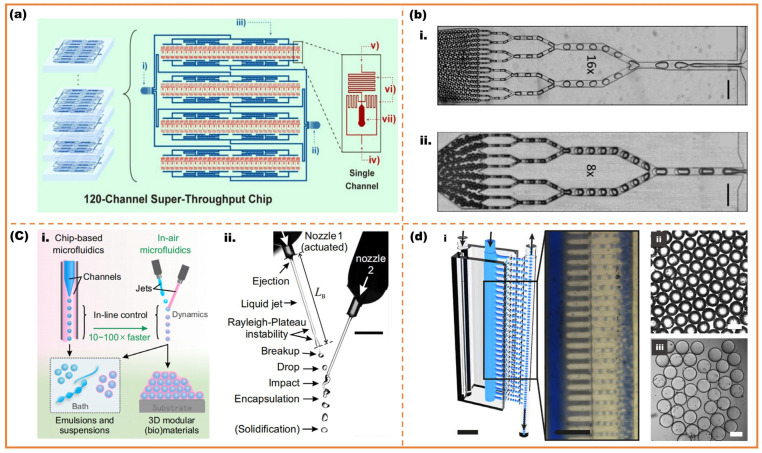
Scaled-up droplet generation with unordinary methods; (**a**) geometries of the 120 flow-focusing units: (**i**) dispersed phase inlet; (**ii**) continuous phase inlet; (**iii**) equal-length inlet channel; g; (**iv**) continuous phase inlet; (**v**) dispersed phase inlet; (**vi**) damping structure for fluid stabilizing; (**vii**) droplet outlet [95]; (**b**) the fabrication of single and double emulsion with droplet splitting method: (**i**) splits the drops four times, into 16 equal portions; (**ii**) splits only three times, into 8 equal portions [99]; (**c**) ‘in-air’ microfluidics: (**i**) schematic diagram of chip-based microfluidics and ‘in-air’ microfluidics; (**ii**) high-speed photograph of the ‘in-air’ device [100]; (**d**) 3D 28 parallel flow-focusing units scaled-up microfluidics: (**i**) schematic diagram and optical images of 28 parallel flow-focusing units droplet generation; (**ii**) optical photograph of monodisperse water droplets; (**iii**) optical photograph of microgels in aqueous medium [101].

Sun et al. manufactured monodisperse microspheres with 120 flow-focusing units on a chip which achieved the high throughput with 109 microspheres per hour, as shown in Figure 8a [95]. Furthermore, another way to realize scaling-up microfluidic fabrication is high-throughput technology based on continuous splitting. The bigger single droplet can be divided into multiple droplets by the shear of specific geometric structures to improve the droplet productivity [96,97,98]. Abate et al. utilized splitting arrays for the production of monodisperse emulsions at high throughput [99]. The devices can split droplets 4 times for single emulsion and 3 times for double emulsion, fabricating 16 and 8 equal portions respectively, as shown in Figure 8b. Visser et al. fabricated monodisperse emulsions through ‘in-air’ microfluidics at rates 10 to 100 times than the droplets generation on chips, as shown in Figure 8c [100]. Femmer et al. prepared a scaled-up microsphere device with 28 parallel flow-focusing units, which produced single emulsion droplet with the rate of 3 L/h, as shown in Figure 8d [101]. High-throughput technology not only offers precise control through droplet microfluidic method but also ensures high output and continuity. It has a wide range of applications in the large-scale industrialization of controllable size PCM microcapsules.

## 3. Energy Storage Application

The high energy storage density and the isothermal quality are the main reasons why the latent heat storage system based on phase-change materials is an effective way of storing thermal energy [102]. As a much more robust form of PCM, phase-change microcapsules can more effectively complete the application and development of energy storage.

**Figure 9:**
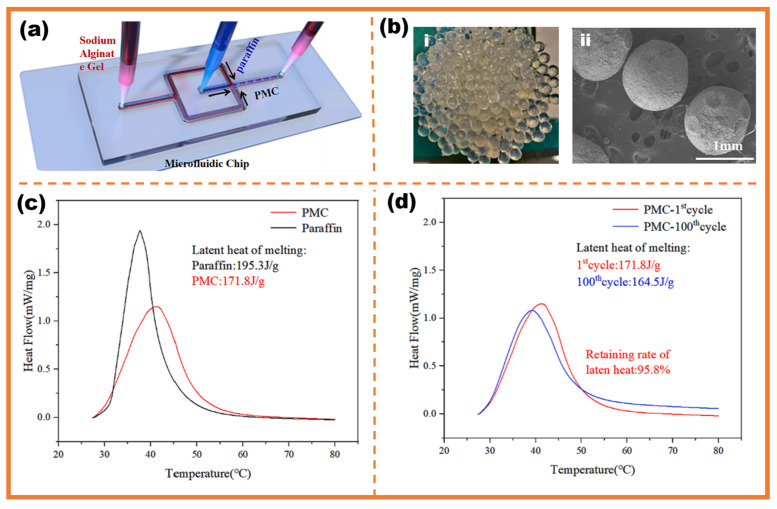
(**a**) Schematic diagram of flow-focusing microfluidic fabricated microcapsules; (**b**): (**i**) phase-change microcapsules samples; (**ii**) SEM images of paraffin phase-change microcapsules; (**c**) DSC curves of paraffin phase-change microcapsules and paraffin; (**d**) DSC curves of paraffin phase-change microcapsules for the 1st and 100th melting-freezing cycles [103].

With conventional energy sources facing constant depletion and rising demand, the advantages of phase-change microcapsules in the solar energy storage field have gained significant attention. Hu et al. utilized the flow-focusing droplet microfluidic method to develop a phase-change microcapsule for solving the challenge of liquid leakage in solar energy storage, as shown in Figure 9a,b. The differential scanning calorimetry (DSC) measurements show that compared with unpackaged PCMs, the phase-change microcapsules remain over 88% latent heat (up to 171.8 J/g), as shown in Figure 9c. Through 100 consecutive melting-freezing cycles with no leakage, they exhibit the stability of microcapsule encapsulation. The unique structure of microencapsulation allows for a wider range of combinations of modified materials and PCMs to enhance the ability of solar energy storage, as shown in Figure 9d [103]. Huang et al. encapsulated eicosane (PCM) and PMMA-modified BPs (mBPs) together to form microcapsules which exhibited a high latent heat of 180 kJ/kg, excellent thermal reliability and photothermal characteristics inherited from BPs. Microcapsules with mPBs integrated into the core exhibited three times higher efficiency in solar energy storage compared to those mPBs on the surface [104]. Sun et al. fabricated microcapsules based on n-octadecane cores and SiO_2_ shells as main materials. The microcapsules with boron nitride (BN) anchored on the surface of SiO_2_ shells exhibited good heat storage performance of 140.6 J/g, along with enhanced heat transfer performance and photothermal conversion efficiency of 69.54% [105]. Liu et al. microencapsulated n-tetracosane and n-eicosane as PCM cores with SiO_2_/Fe_3_O_4_ composite shells along with surface-decorated MXene nanosheets which obviously achieved high light absorption efficiency of 95.4% [106]. Su et al. fabricated a novel phase-change microcapsule (O/W, paraffin wax as core, melamine-formaldehyde (MF) as shell) for solar-assisted hot water storage system [107]. The analysis revealed that the five MF samples exhibited favorable enthalpy values, particularly MF-3, which demonstrated the highest enthalpy of 126 kJ/kg. This makes it suitable for solar-assisted hot water storage systems, offering higher energy storage density and a relatively smaller physical storage size compared to water-based systems.

With the deepening of research, thermal energy storage microcapsules, especially photo-thermal energy storage phase-change microcapsules, have been developed combined with energy efficient buildings, temperature-controlled textiles, seawater desalination and other fields [108]. The fabrication method of microfluidic is still waiting to explore and scale up.

## 4. Thermal Regulation Application

The thermal regulation is the most well-known application of phase-change microcapsules for its homeothermy, which can maintain a safe range of temperature [109,110]. Freezing and heating are the primary application scenarios in which thermal regulation is utilized [24].

### 4.1. Biulding

Temperature variations occur in buildings due to heat sources such as machinery, computer facilities, and human occupancy [111]. Reducing power loss in room heating and cooling has been a key challenge. Phase-change microcapsules as a selection used for application in buildings depend on their thermophysical properties [112]. The optimal selection standard of PCM for building applications are appropriate phase transition temperature, stable phase transition, appropriate thermo-physical properties, low cost, nontoxic, non-flammable, and recyclable [113]. The composite PCMs have good thermal properties and can be properly used as latent heat materials for indoor temperature regulation and energy storage [114,115,116]. The phase-change microcapsules effectively adapt its thermophysical properties. Furthermore, its shell–core structure isolates the internal environment from the external one, thus effectively addressing issues related to toxicity, flammability, and corrosiveness. The most common way to utilize the phase-change microcapsules in building fields is to blend them into cement and slurry.

Cao et al. mixed phase-change microcapsules into Portland cement concrete (PCC) and geopolymer concrete (GPC) leading to concretes with both effect of thermal performance from phase-change microcapsules and compressive strength properties from PCC and GPC [117]. With the phase-change microcapsules replacing the sand, the thermal conductivity of concrete reduced and the thermal energy storage increased, while the heat capacity of concrete maintained a stable range during the phase in the condition of liquid and solid. Dutkowski et al. measured the thermal conductivity of the composites of aqueous slurry and phase-change microcapsules, which contained about 43% capsulated paraffin [118]. The study showed the thermal conductivity about composites with different contents about phase-change microcapsules with a transformation temperature of 28 °C [119].

Adding phase-change microcapsules to window and wall materials can effectively reduce heat loss, decrease the frequency of heating and cooling system use, and achieve energy-saving and emission reduction goals. Zhang et al. incorporated phase-change microcapsules into Al_2_O_3_-SiO_2_ aerogels, which was called phase-change microcapsule with ASA to improve thermal regulation properties [120]. The phase-change microcapsule with ASA revealed 4.1wt% shrinkage, 380.22 m^2^/g specific surface area, and 0.0507 W/(m·K) thermal conductivity at room temperature, which enhanced heat conversion rates in building insulation materials, demonstrating significant potential in thermal energy storage and regulation. Long et al. fabricated a novel superhydrophobic coating which incorporated phase-change microcapsules into polydimethylsiloxane [121]. The coating reflected more than 90% solar energy and exhibited passive daytime radiative cooling temperature of 13.07 °C in summer and 5.54 °C in autumn. The composite coating reduced indoor temperatures without additional energy input. This technology shows promise for passive cooling applications in buildings.

Phase-change microcapsules have broad application prospects in the construction field, and preparing phase-change microcapsules through microfluidic methods is an important direction for future development. Considering the huge demand for microcapsule materials in the construction industry, the methodology for size-controlled microcapsule fabrication using high-throughput microfluidics is poised to become a key area for future development.

### 4.2. Electronic Equipment

With the advancement of automation and informatization technology across various industries, the demand for electronic equipment has significantly increased. Effective thermal management is a crucial factor in determining the service life of these devices. Generally, thermal management in electronic equipment is categorized into active and passive methods [122]. Active thermal management in electronic devices refers to dynamically adjusting the internal temperature of the device to ensure its performance and longevity. Major methods include the use of fans, liquid cooling systems, thermoelectric coolers etc. [123]. However, active thermal management methods are more complex than passive method, which makes them more prone to system failures and noise issues, ultimately leading to increased costs.

Phase-change microcapsules contain phase-change materials which can absorb or release heat at specific phase transition temperatures. When electronic devices operate, they generate substantial heat, leading to an increase in internal temperature. The phase-change material within the microcapsules absorbs the heat and undergoes a phase change, thereby mitigating the rate of temperature rise. Conversely, when the device temperature decreases, the phase-change material releases the previously stored heat, maintaining the device within a stable temperature range. The phase-change temperature control technology, initially developed for spacecraft, has been adapted for electronic devices [124]. Alshaer et al. employed carbon foam (CF) combined with paraffin wax (RT65) as a phase-change material to build a hybrid composite system for thermal management of electronic devices [125]. The experimental study showed CF with RT65 had a reasonable delay for reaching temperature stability compared to pure CF. Currently, electronic devices are evolving towards greater portability and miniaturization. Phase-change microcapsules offer distinct advantages in the thermal management of micro-electronic devices.

**Figure 10:**
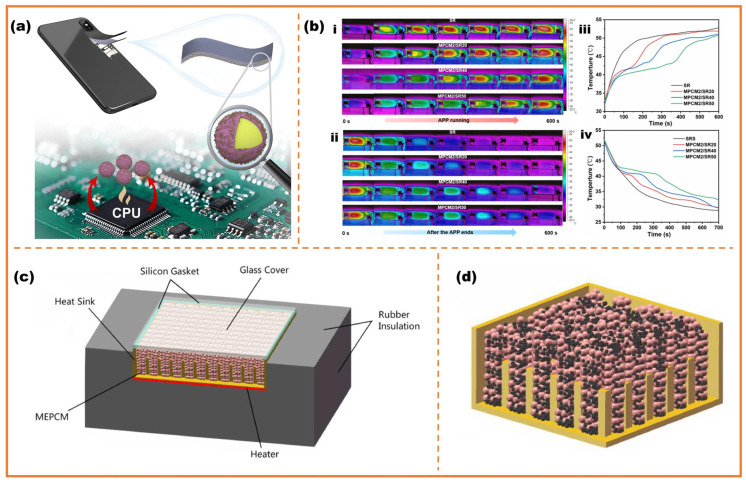
(**a**) Schematic illustration showing the preparation and application of the RCh/PU phase-change microcapsules; (**b**): (**i**) infrared thermal images with APP running; (**ii**) infrared thermal images after the APP ends; (**iii**) the corresponding time histories of surface temperatures with APP running; (**iv**) the corresponding time histories of surface temperatures after APP ends [126]. (**c**) schematic diagram of phase-change microcapsules heat sink assembly; (**d**) schematic diagram of sink filled with phase-change microcapsules-EG composite with 25 pin fins of 9 mm^2^ cross section [127].

Zhu et al. utilized paraffin wax as cores and polyurea as shells for mobile devices [126]. The phase-change microcapsules were investigated to delay temperature rise against high processor demands in scenarios like multitasking, as shown in Figure 10a. Ren et al. employed commercial paraffin with volume fraction of 61.663% as cores and the calcium carbonate (CaCo_3_) with volume fraction of 38.37% as shells to fabricate phase-change microcapsules [127]. The group mixed phase-change microcapsules with expanded graphite to fill into the heat sink assembly and carry out a numerical study with different pin-fins, EG content, PCM melting temperature and heat flux conditions. The result showed phase-change microcapsules-EG composite materials enhanced the heat absorption through latent energy absorption.

### 4.3. Textile

The body temperature as a common constant temperature application circumstance can be well adapted to the phase-change materials for their homeothermy. Phase-change microcapsules’ advantages in PCM packaging enable it to better adapt to the textile. The microcapsules have a wide range of substances and robust properties of thermal and structural mechanics [127,128]. Maintaining a comfortable and safe body temperature is the main application of phase-change microcapsules in textiles [129].

**Figure 11:**
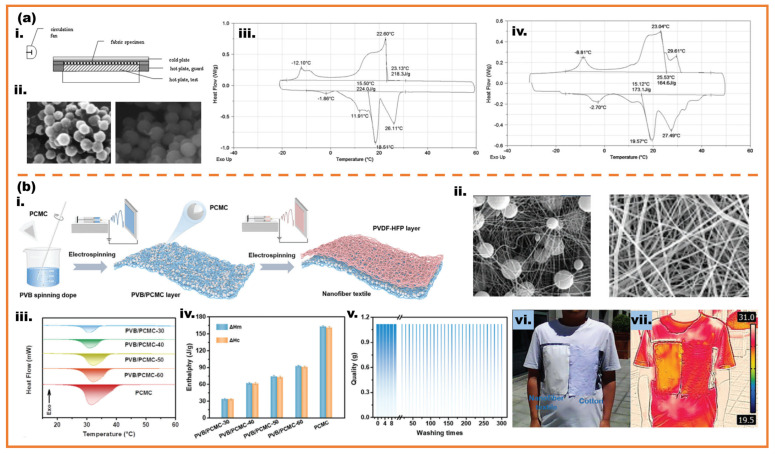
(**a**): (**i**) schematic diagram of guarded hot plate apparatus; (**ii**) SEM micrographs of microcapsules; (**iii**) DSC thermograms of PCMs microcapsules of n-hexadecane/n-eicosane binary mixture; (**iv**) DSC thermograms of PCMs microcapsules of encapsulated PCMs [130]. (**b**): (**i**) schematic diagram of preparation process of the nanofiber textile; (**ii**) SEM micrographs of microcapsules and fibers; (**iii**) DSC heating curves with different amounts of phase-change microcapsules; (**iv**) melting enthalpy and crystallization enthalpy with different amounts of phase-change microcapsules; (**v**) washability of the nanofiber textile; (**vi**,**vii**) visible and infrared thermography photos of the nanofiber textile and cotton on the human chest at thermal stabilization [133].

Salaün et al. fabricated melamine-formaldehyde microcapsules containing n-alkane mixtures for thermoregulating textile fabrics, as shown in Figure 11a [130]. The result by DSC showed that the microcapsules allowed to delay the temperature increase. Shin et al. prepared microcapsules with the structure of melamine-formaldehyde as the shell and eicosane as the core. The thermoregulating fabrics combined with microcapsules showed heat storage capacities of 0.91–4.44 J/g [131]. Zhang et al. fabricated microcapsules with the solid paraffin and butyl stearate as core and methyl methacrylate as monomer and attached by the SiO_2_ microparticles [132]. The phase-change microcapsules were applied to the denim fabric, effectively reducing the cooling rate of the outer fabric. This treatment decelerated heat dissipation and prolonged the duration of heat release. Gu et al. proposed an approach for fabricating hierarchical nanofiber textiles embedded with phase-change microcapsules via electrospinning, as shown in Figure 11b. The results demonstrate that the composite fibers achieve a high phase-change enthalpy of 92.6 J/g, significantly enhancing thermal buffering capacity. Remarkably, the textile maintains structural integrity without leakage after 300 washing cycles and 100 phase-change cycles [133].

Currently, thermal control fabrics are produced through impregnation or surface coating techniques. Phase-change microcapsules are utilized in garments such as underwear, sweaters, and coats due to their effective temperature regulation properties. However, the preparation of phase-change microcapsules is complex and costly, and their limited moisture permeability, among other issues, poses significant challenges to large-scale application [134,135].

## 5. Conclusions and Challenge

Microfluidics has fundamentally redefined phase-change microcapsule engineering, providing remarkable control over particle morphology, encapsulation efficiency, and functional design. By enabling precise control over microscale droplet, core–shell architectures, and encapsulation efficiency, microfluidic techniques (including single/multiple emulsion encapsulation and high-throughput parallelization) overcome inherent limitations of conventional fabrication. These engineered PCMs demonstrate exceptional versatility in critical applications: solar energy storage (resolving leakage challenges), building thermal management (enhancing cementitious composites), electronics cooling (delaying temperature spikes), and smart textiles (maintaining thermal comfort). The continuous refinement of microfluidic strategies has established a robust platform for designing next-generation phase-change materials with customized properties, driving innovation in thermal energy regulation technologies across multiple disciplines, but significant challenges persist:

(1) Material innovation: current PCMs (e.g., n-alkanes) exhibit limited phase-change enthalpy and thermal stability. Future research should prioritize developing novel PCM composites (e.g., eutectic mixtures, metal-enhanced formulations) with higher latent heat, tailored phase transition temperatures, and improved cyclability. These materials have to maintain stability under repeated thermal cycles while minimizing supercooling and phase separation;
(2) Fabrication techniques:
  - Sub-micron Particles: Downsizing microcapsules to sub-micron scales (<1 μm) could enhance heat transfer kinetics but complicates production. Current microfluidic methods achieve monodispersity at scales of 10–1000 μm and scaling down may compromise throughput.
  - Particle Grading: Monodisperse microcapsules limit packing density in composites (e.g., concrete, slurries). Implementing size-graded distributions (e.g., bimodal/multimodal) during microfluidic fabrication is able to optimize interstitial filling, increasing volumetric enthalpy and thermal conductivity in end-use applications.
(3) High-throughput scalability: While parallelized microfluidics (e.g., kilo-scale flow-focusing arrays, step emulsifiers) boosts droplet production rates to liters per hour, maintaining low size dispersity (CV < 5%) remains challenging at industrial scales. Key bottlenecks include inherent inter-branch flow variations within complex distribution networks, leading to non-uniform shear stress and droplet polydispersity. Furthermore, the extended operation necessary for high throughput exacerbates the risk of chip flow channel clogging, particularly in sensitive or complex emulsion systems (e.g., high viscosity, particle-laden, or multi-phase formulations) where material accumulation at junctions, bends, or low-flow zones becomes problematic. Future designs must integrate intelligent flow distribution networks incorporating real-time pressure and flow sensors with closed-loop active control. This approach dynamically adjusts branch resistances to ensure uniform volumetric flow and shear profile across all parallel units, thereby minimizing droplet size dispersity. Concurrently, robust anti-clogging strategies are essential, which includes: (1) optimizing channel geometry through low-volume, low-tortuosity designs with appropriate surface treatments; (2) integrating localized actuation mechanisms (e.g., piezoelectric pulsations, thermal elements, or integrated bypass flushing ports) at critical nodes to dislodge nascent blockages without interrupting production.

## Figures and Tables

**Table 1:** Thermal properties of common phase-change materials [6,8,17,18,19,20].

Material	Temperature (°C)	Thermal Conductivity (W/(mK))	Phase-Change Enthalpy (kJ/kg)
Molten salts (NaNO_3_-KNO_3_-NaNO_2_)	142	0.60	75–84
Natural Ester Oil	−25	0.16–0.23	150–220
n-Dodecane (C_12_H_26_)	−12	0.21 (s), 0.21 (l)	216
n-Pentadecane (C_15_H_32_)	10	0.17	207
n-Hexadecane (C_16_H_34_)	18.2	0.21 (s)	238
n-Octadecane (C_18_H_38_)	28.2	0.35 (s), 0.149 (l)	245
n-Nonadecane (C_19_H_40_)	31.9	0.21 (s)	222

## Abbreviations

PCM	Phase-change material
TES	Thermal energy storage
SHS	Sensible heat storage
LHS	Latent heat storage
ppm	Parts per million
PMMA	Polymethyl-methacrylate
PDMS	Polydimethylsiloxane
W/O	Water-in-oil
O/W	Oil-in-water
CV	Coefficient of variation
W/O/W	Water-in-oil-in-water
O/W/O	Oil-in-water-in-oil
UV	Ultraviolet
n.a.	Not available
DSC	Differential scanning calorimetry
SEM	Scanning electron microscopy
PCC	Portland cement concrete
GPC	Geopolymer concrete
wt%	Weight percent
CF	Carbon foam
EG	Expanded graphite
MF	Melamine-formaldehyde

## References

[1] Bi C., Li G. (2024). Analysis of Problems and Countermeasures in the Promotion of New Energy Vehicles in China. Trans. Econ. Bus. Manag. Res..

[2] Raimi D., Newell R., Prest B., Villanueva S., Wingenroth J. (2024). Global Energy Outlook 2024: Peaks or Plateaus.

[3] Sarbu I., Sebarchievici C. (2018). A Comprehensive Review of Thermal Energy Storage. Sustainability.

[4] Gil A., Medrano M., Martorell I., Lázaro A., Dolado P., Zalba B., Cabeza L.F. (2010). State of the Art on High Temperature Thermal Energy Storage for Power Generation. Part 1—Concepts, Materials and Modellization. Renew. Sustain. Energy Rev..

[5] Boshell F., Vela I., Visscher K. (2020). Innovation Outlook: Thermal Energy Storage.

[6] Alva G., Lin Y., Fang G. (2018). An Overview of Thermal Energy Storage Systems. Energy.

[7] Hasnain S.M. (1998). Review on Sustainable Thermal Energy Storage Technologies, Part I: Heat Storage Materials and Techniques. Energy Convers. Manag..

[8] Li S., Li J., Feng Y., Zhao Y., Zhao Y., Ding H., Gong S. (2020). A Review on Thermal Conductivity of Magnesium and its Alloys. J. Magnes. Alloys.

[9] Zhan H., Lin G., Li Y. (2023). A Case Study on Energy Conservation Using Building Performance Simulation (BPS) to Validate and Analyse a Phase Change Material (PCM) Integrated Office Building. SSRN Online.

[10] Alrashdan A., Kharrufa S., Ahmed A. (2024). Integration of Phase Change Materials in Service Areas of Building Envelopes for Improved Thermal Performance: An Experimental Study in Saudi Arabia. Buildings.

[11] Franzén B., Auer G., Lewensohn R. (2024). Minimally Invasive Biopsy-Based Diagnostics in Support of Precision Cancer Medicine. Mol. Oncol..

[12] Yuan K., Pan Y., Zhang X., Zhang X., Yin H., Chen W., Gao W. (2024). Micro/Nano Encapsulated Phase Change Materials: Preparation, Principle, and Emerging Advances in Medical Field. Adv. Funct. Mater..

[13] Mohamed H.A., El-Hadidy H., El-Dessouky H.M. (2025). Microencapsulation and its Application in Textile Industry. J. Text. Color. Polym. Sci..

[14] Wang X., Jiang H., Liang S., Yang C., Zhang P. (2024). Experimental Study on the Thermal Protection Enhancement of Novel Phase Change Material Integrated Structural Firefighting Gloves Under High-Heat Exposures. Case Stud. Therm. Eng..

[15] Li M., Liu S. (2024). Metal-Polyphenol Based Phase Change Microcapsules for Photothermal Conversion and Storage. Sol. Energy Mater. Sol. Cells.

[16] Murali G., Arjunan T.V., Shahapurkar K., Tirth V., Soudagar M.E.M., Khan T.M.Y., Elfasakhany A. (2024). Experimental Studies on Solar Reusable Can Air Heating System Integrated with Latent Heat Storage. J. Therm. Anal. Calorim..

[17] Huang J., Zhang Y., Ding Y., Guo X., Zhou H. (2021). Advances and Applications of Phase Change Materials (PCMs) and PCMs-Based Technologies. ES Mater. Manuf..

[18] Su W., Darkwa J., Kokogiannakis G. (2015). Review of Solid–Liquid Phase Change Materials and their Encapsulation Technologies. Renew. Sustain. Energy Rev..

[19] Lane G.A. (1983). Solar Heat Storage: Latent Heat Materials.

[20] Sharma S.D., Kitano H., Sagara K. (2004). Phase Change Materials for Low Temperature Solar Thermal Applications. Res. Rep. Fac. Eng. Mie Univ..

[21] Wu Y., Chen M., Zhao G., Qi D., Zhang X., Li Y., Huang Y., Yang W. (2024). Recyclable solid–solid phase change materials with superior latent heat via reversible anhydride-alcohol crosslinking for efficient thermal storage. Adv. Mater..

[22] Geng X., Hu Y., Pan H., Wang C., Liu Z., He X. (2024). Biodegradable polylactic acid/polyethylene glycol blends as form-stable phase change materials for thermal energy storage and management. Polymer.

[23] Guo Y., Zhao J., Xu X., Zhang Y., Gao X., Zhang H., Wang H. (2023). Phase Change Materials Meet Microfluidic Encapsulation. Adv. Sci..

[24] Gao W., Zhao L., Cheng C., Liang S., Wu J., Lin Z., Fu J. (2023). Microfluidic Method–Based Encapsulated Phase Change Materials: Fundamentals, Progress, and Prospects. Renew. Sustain. Energy Rev..

[25] Mateovic T., Kriznar B., Bogataj M., Mrhar A. (2002). The Influence of Stirring Rate on Biopharmaceutical Properties of Eudragit RS Microspheres. J. Microencapsul..

[26] Kadam N.R., Suvarna V. (2015). Microsphere: A Brief Review. Asian J. Biomed. Pharm. Sci..

[27] Ho Y.K., Choi Y.H., Wiley D.E. (2004). Preparation of Alginate Microspheres Using Membrane Emulsification Method. Membr. J..

[28] Guha K., Rani M.U., Sarma S. (2022). MEMS and Microfluidics in Healthcare. Microfluidics and Bio-MEMS: Devices and Applications.

[29] Juang Y.J., Chiu Y.J. (2022). Fabrication of Polymer Microfluidics: An Overview. Polymers.

[30] Manz A., Graber N., Widmer H.M. (1990). Miniaturized Total Chemical Analysis Systems: A Novel Concept for Chemical Sensing. Sens. Actuators B Chem..

[31] Riahi R., Tamayol A., Shaegh S.A.M., Ghaemmaghami A.M., Dokmeci M.R., Khademhosseini A. (2015). Microfluidics for Advanced Drug Delivery Systems. Curr. Opin. Chem. Eng..

[32] Mu X., Zhang H., Wang L., Gu W., Gall K. (2013). Microfluidics for Manipulating Cells. Small.

[33] Huang L., Chen Y., Feng Y., He Z., Shen Y. (2021). One-Step Microfluidic Synthesis of Spherical and Bullet-Like Alginate Microcapsules with a Core–Shell Structure. Colloids Surf. A Physicochem. Eng. Asp..

[34] Cramer C., Fischer P., Windhab E.J. (2004). Drop Formation in a Co-Flowing Ambient Fluid. Chem. Eng. Sci..

[35] Thorsen T., Roberts R.W., Arnold F.H., Quake S.R. (2001). Dynamic Pattern Formation in a Vesicle-Generating Microfluidic Device. Phys. Rev. Lett..

[36] Garstecki P., Fuerstman M.J., Stone H.A., Whitesides G.M. (2006). Formation of Droplets and Bubbles in a Microfluidic T-Junction—Scaling and Mechanism of Break-Up. Lab Chip.

[37] Anna S.L., Bontoux N., Stone H.A. (2003). Formation of Dispersions Using “Flow Focusing” in Microchannels. Appl. Phys. Lett..

[38] Dreyfus R., Tabeling P., Willaime H. (2003). Ordered and Disordered Patterns in Two-Phase Flows in Microchannels. Phys. Rev. Lett..

[39] Haeberle S., Zengerle R. (2007). Microfluidic Platforms for Lab-on-a-Chip Applications. Lab Chip.

[40] Qin D., Xia Y., Whitesides G.M. (2010). Soft Lithography for Micro- and Nanoscale Patterning. Nat. Protoc..

[41] Xu J.H., Li S.W., Tan J., Luo G.S. (2006). Controllable Preparation of Monodisperse O/W and W/O Emulsions in the Same Microfluidic Device. Langmuir.

[42] Trujillo-Cayado L.A., Ramírez P., Alfaro M.C., Muñoz J. (2016). A Further Step in the Development of Oil-in-Water Emulsions Formulated with a Mixture of Green Solvents. Ind. Eng. Chem. Res..

[43] Dao T.D., Jeong H.M. (2015). Novel Stearic Acid/Graphene Core–Shell Composite Microcapsule as a Phase Change Material Exhibiting High Shape Stability and Performance. Sol. Energy Mater. Sol. Cells.

[44] Dai Z., Zhang X., Wang L., Chen Y., Chen Q. (2025). Microfluidic-Assisted Sol–Gel Preparation of Monodisperse Mesoporous Silica Microspheres with Controlled Size, Surface Morphology, Porosity and Stiffness. Nanoscale.

[45] Chen S., Shahar T., Cohen S. (2024). Thermo-Controlled Microfluidic Generation of Monodisperse Alginate Microspheres Based on External Gelation. RSC Adv..

[46] Zheng Y., Zhang Y., Li J., Liu X., Liu Z. (2025). High-Throughput Preparation of Monodisperse Biocompatible Core-Shell Capsules by 3D-Printed Microfluidics. Chem. Eng. Sci..

[47] Tahan Latibari S., Mehrali M., Mehrali M., Mahlia T.M.I., Metselaar H.S.C. (2015). Fabrication and Performances of Microencapsulated Palmitic Acid with Enhanced Thermal Properties. Energy Fuels.

[48] Lone S., Chew S.F., van der Walle C.F. (2013). Facile and Highly Efficient Microencapsulation of a Phase Change Material Using Tubular Microfluidics. Colloids Surf. A Physicochem. Eng. Asp..

[49] Hao G., Dong H., Zhang Z., Zhou J., Liu Y., Chen Y. (2022). Controlled Microfluidic Encapsulation of Phase Change Material for Thermo-Regulation. Int. J. Heat Mass Transf..

[50] Poulsen C.E., Christensen K., Breil M.P., Thybo P., Qvortrup K., Nielsen S.S., Hall V., Hansen S.H., Jensen H. (2015). A Microfluidic Platform for the Rapid Determination of Distribution Coefficients by Gravity-Assisted Droplet-Based Liquid–Liquid Extraction. Anal. Chem..

[51] Zhang Y., Nguyen N.T. (2022). A Perspective on Magnetic Microfluidics: Towards an Intelligent Future. Biomicrofluidics.

[52] Vladisavljević G.T., Al Nuumani R., Nabavi S.A. (2017). Microfluidic Production of Multiple Emulsions. Micromachines.

[53] Utada A.S., Lorenceau E., Link D.R., Kaplan P.D., Stone H.A., Weitz D.A. (2005). Monodisperse Double Emulsions Generated From a Microcapillary Device. Science.

[54] Abate A.R., Weitz D.A. (2009). High-Order Multiple Emulsions Formed in Poly(dimethylsiloxane) Microfluidics. Small.

[55] Nie Z., Li W., Seo M., Xu S., Kumacheva E. (2006). Janus and Ternary Particles Generated by Microfluidic Synthesis: Design, Synthesis, and Self-Assembly. J. Am. Chem. Soc..

[56] Wang W., Zhang M.J., Chu L.Y. (2014). Microfluidic Approach for Encapsulation via Double Emulsions. Curr. Opin. Pharmacol..

[57] Nurumbetov G., Ballard N., Bon S.A.F. (2012). A Simple Microfluidic Device for Fabrication of Double Emulsion Droplets and Polymer Microcapsules. Polym. Chem..

[58] Gao W., Chen Y. (2019). Microencapsulation of Solid Cores to Prepare Double Emulsion Droplets by Microfluidics. Int. J. Heat Mass Transf..

[59] Wang N., Liu H., Wan J., Chen Y. (2020). Modelling Double Emulsion Formation in Planar Flow-Focusing Microchannels. J. Fluid Mech..

[60] Abate A.R., Kutsovsky M., Seiffert S., Windberg M., Rotem A., Weitz D.A. (2011). Synthesis of Monodisperse Microparticles from Non-Newtonian Polymer Solutions with Microfluidic Devices. Adv. Mater..

[61] Lee T., Liu L. (2010). Lattice Boltzmann Simulations of Micron-Scale Drop Impact on Dry Surfaces. J. Comput. Phys..

[62] Azarmanesh M., Farhadi M., Azizian P. (2016). Double Emulsion Formation Through Hierarchical Flow-Focusing Microchannel. Phys. Fluids.

[63] Lee T.Y., Choi T.M., Shim T.S., Weitz D.A. (2016). Microfluidic Production of Multiple Emulsions and Functional Microcapsules. Lab Chip.

[64] Okushima S., Nisisako T., Torii T., Higuchi T. (2004). Controlled Production of Monodisperse Double Emulsions by Two-Step Droplet Breakup in Microfluidic Devices. Langmuir.

[65] Chen Y., Deng Z. (2017). Hydrodynamics of a Droplet Passing Through a Microfluidic T-Junction. J. Fluid Mech..

[66] Ngo I.L., Khoo B.C., Yang C. (2015). A Numerical Study on the Dynamics of Droplet Formation in a Microfluidic Double T-Junction. Biomicrofluidics.

[67] Nabavi S.A., Vladisavljević G.T., Gu S. (2015). Double Emulsion Production in Glass Capillary Microfluidic Device: Parametric Investigation of Droplet Generation Behaviour. Chem. Eng. Sci..

[68] Akamatsu K., Kaneko Y., Nakayama S., Kikuchi R., Yamada H., Shibata T. (2019). A Facile Microencapsulation of Phase Change Materials within Silicone-Based Shells by Using Glass Capillary Devices. Colloids Surf. A Physicochem. Eng. Asp..

[69] Han X., Shen Y., Guo X., Zhang Y. (2020). Microfluidic Encapsulation of Phase-Change Materials for High Thermal Performance. Langmuir.

[70] Wang C., Gao W., Chen Y., Fu J. (2022). Fabrication of Microencapsulated Phase Change Materials Using Microfluidics. Proceedings of the MEMAT 2022; 2nd International Conference on Mechanical Engineering, Intelligent Manufacturing and Automation Technology, Guilin, China, 7–9 January 2022.

[71] Nisisako T., Hatsuzawa T. (2016). Microfluidic fabrication of oil-filled polymeric microcapsules with independently controllable size and shell thickness via Janus to core–shell evolution of biphasic droplets. Sens. Actuators B Chem..

[72] Yao X., Zhang Y., Du L., Liu J., Yao J. (2015). Review of the Applications of Microreactors. Renew. Sustain. Energy Rev..

[73] Gale B.K., Jafek A.R., Lambert C.J., Goenner B.L., Moghimifam H., Nze U.C., Kamande S.M. (2018). A Review of Current Methods in Microfluidic Device Fabrication and Future Commercialization Prospects. Inventions.

[74] Mulligan M.K., Rothstein J.P. (2012). Scale-Up and Control of Droplet Production in Coupled Microfluidic Flow-Focusing Geometries. Microfluid. Nanofluid..

[75] Liu X., Xu J., Wu J., Guo Y. (2024). High-Throughput Microfluidic Production of Carbon Capture Microcapsules: Fundamentals, Applications, and Perspectives. Int. J. Extreme Manuf..

[76] Su Y.Y., Tan B.T., Chen C.H. (2024). Simple and Flexible Production of Controllable Emulsion Droplets from Open-Type Co-Flow Microfluidics. Chem. Eng. Sci..

[77] Chen X., Li T., Zeng H., Hu Z., Fu X. (2015). Model of Droplet Generation in Flow Focusing Generators Operating in the Squeezing Regime. Microfluid. Nanofluid..

[78] Gupta A., Murshed S.M.S., Kumar R. (2009). Droplet Formation and Stability of Flows in a Microfluidic T-Junction. Appl. Phys. Lett..

[79] Eggersdorfer M.L., Seybold H., Ofner A., Weitz D.A., Studart A.R. (2018). Wetting Controls of Droplet Formation in Step Emulsification. Proc. Natl. Acad. Sci. USA.

[80] Han T., Wei J., Li Z., Gao Z., Xu Y., Zhang W. (2017). Factory-on-Chip: Modularised Microfluidic Reactors for Continuous Mass Production of Functional Materials. Chem. Eng. J..

[81] Conchouso D., Carreno A.A.A., Castro D., Khan S.A., Foulds I.G. (2014). Three-Dimensional Parallelization of Microfluidic Droplet Generators for a Litre per Hour Volume Production of Single Emulsions. Lab Chip.

[82] Huang Y., Zhang Y., Sun H., Li J., Zhang X., Zhu P. (2018). Design Criteria and Applications of Multi-Channel Parallel Microfluidic Module. J. Micromech. Microeng..

[83] Hashimoto M., Shevkoplyas S.S., Zason B., Szymborski T., Garstecki P., Whitesides G.M. (2008). Formation of Bubbles and Droplets in Parallel, Coupled Flow-Focusing Geometries. Small.

[84] Wu J., Kong T., Yeung K.W.K., Shum H.C., Lam R.H.W. (2021). Scaling Up the Throughput of Microfluidic Droplet-Based Materials Synthesis: A Review of Recent Progress and Outlook. Appl. Phys. Rev..

[85] Romanowsky M.B., Abate A.R., Rotem A., Holtze C., Weitz D.A. (2012). High Throughput Production of Single Core Double Emulsions in a Parallelized Microfluidic Device. Lab Chip.

[86] Yi H., Wang F., Li Y., Zhang H., Qin S., Li S., Hu Z. (2022). Parallelized Microfluidic Droplet Generators with Improved Ladder–Tree Distributors for Production of Monodisperse γ-Al_2_O_3_ Microspheres. Particuology.

[87] Håti A.G., Morch Y.A., Røe R., Dyrstad K., Sæther B., Angell-Petersen E., Furuseth K., Skotland T., Sandvig K., Sioud M. (2018). Production of Monodisperse Drops from Viscous Fluids. Lab Chip.

[88] Vladisavljević G.T., Kobayashi I., Nakajima M., Williams R.A., Shono A., Satoh K. (2018). Long-Term Stability of Droplet Production by Microchannel (Step) Emulsification in Microfluidic Silicon Chips with Large Number of Terraced Microchannels. Chem. Eng. J..

[89] Li W., Greener J., Voicu D., Kumacheva E. (2009). Multiple Modular Microfluidic (M-3) Reactors for the Synthesis of Polymer Particles. Lab Chip.

[90] Tetradis-Meris G., Rossetti D., de Torres C.P., Cao R., Ianoul P., Lian G., Janvier P. (2009). Novel Parallel Integration of Microfluidic Device Network for Emulsion Formation. Ind. Eng. Chem. Res..

[91] Jeong H.H., Yelleswarapu V.R., Yadavali S., Issadore D., Lee D. (2015). Kilo-Scale Droplet Generation in Three-Dimensional Monolithic Elastomer Device (3D MED). Lab Chip.

[92] Nisisako T., Torii T. (2008). Microfluidic Large-Scale Integration on a Chip for Mass Production of Monodisperse Droplets and Particles. Lab Chip.

[93] Amstad E., Chen D., Zhao M.X., Kim S.H., Weitz D.A. (2016). Robust Scalable High Throughput Production of Monodisperse Drops. Lab Chip.

[94] Stolovicki E., Ziblat R., Weitz D.A. (2018). Throughput Enhancement of Parallel Step Emulsifier Devices by Shear-Free and Efficient Nozzle Clearance. Lab Chip.

[95] Sun K., Zhang X., Liu J., Liu Z., Tan T.T.Y., Sun B. (2024). Microfluidic Precision Manufacture of High Performance Liquid Chromatographic Microspheres. Angew. Chem. Int. Ed..

[96] Hoang D.A., Portela L.M., Kleijn C.R., Kreutzer M.T., Van Steijn V. (2014). Design and Characterization of Bubble-Splitting Distributor for Scaled-Out Multiphase Microreactors. Chem. Eng. J..

[97] Kim C.M., Kim G.M. (2019). Fabrication of 512-Channel Geometrical Passive Breakup Device for High-Throughput Microdroplet Production. Micromachines.

[98] Chen Y., Li G., Borowsky L., Koolivand A., Lian Q. (2016). Three-Dimensional Splitting Microfluidics. Lab Chip.

[99] Abate A.R., Weitz D.A. (2011). Faster Multiple Emulsification with Drop Splitting. Lab Chip.

[100] Visser C.W., Kamperman T., Karbaat L.P., Lohse D., Karperien M. (2018). In-Air Microfluidics Enables Rapid Fabrication of Emulsions, Suspensions, and 3D Modular (bio)materials. Sci. Adv..

[101] Femmer T., Kuehne A.J.C., Wessling M. (2015). High-Throughput Generation of Emulsions and Microgels in Parallelized Microfluidic Drop-Makers Prepared by Rapid Prototyping. ACS Appl. Mater. Interfaces.

[102] Sharma A., Tyagi V.V., Chen C.R., Buddhi D. (2009). Review on Thermal Energy Storage with Phase Change Materials and Applications. Renew. Sustain. Energy Rev..

[103] Hu H., Zhang H., Hong S. (2023). Form-Stable Microencapsulated Phase Change Materials for Efficient Solar Thermal Energy Storage. Mater. Lett..

[104] Huang H., Wei Y., Wang Y., Zhao P., Li X. (2020). Phase-Changing Microcapsules Incorporated with Black Phosphorus for Efficient Solar Energy Storage. Adv. Sci..

[105] Sun J., Ding H., Chen J., Cao L., Yang L. (2022). Biodegradable Wood Plastic Composites with Phase Change Microcapsules of Honeycomb-BN-Layer for Photothermal Energy Conversion and Storage. Chem. Eng. J..

[106] Liu H., Tao X., Wu S., Ye Q. (2023). MXene-Decorated Magnetic Phase-Change Microcapsules for Solar-Driven Continuous Seawater Desalination with Easy Salt Accumulation Elimination. Chem. Eng. J..

[107] Su W., Darkwa J., Kokogiannakis G. (2017). Development of Microencapsulated Phase Change Material for Solar Thermal Energy Storage. Appl. Therm. Eng..

[108] Tan C., He Y., Luo B., Liu M. (2023). Microencapsulated Phase Change Material with Chitin Nanocrystals Stabilized Pickering Emulsion for Thermal Energy Storage. Int. J. Biol. Macromol..

[109] Ma T., Yang H., Lu L. (2015). Using Phase Change Materials in Photovoltaic Systems for Thermal Regulation and Electrical Efficiency Improvement: A Review and Outlook. Renew. Sustain. Energy Rev..

[110] Chandrasekar M., Rajkumar S., Valavan D. (2015). A Review on the Thermal Regulation Techniques for Non Integrated Flat PV Modules Mounted on Building Top. Energy Build..

[111] Rathore P.K.S., Shukla S.K., Gupta N.K. (2020). Potential of Microencapsulated PCM for Energy Savings in Buildings: A Critical Review. Sustain. Cities Soc..

[112] Anisur M.R., Mahfuz M.H., Kibria M.A., Saidur R., Metselaar I.H.S.C., Mahlia T.M.I. (2013). Curbing Global Warming with Phase Change Materials for Energy Storage. Renew. Sustain. Energy Rev..

[113] Konuklu Y., Ostry M., Paksoy H.O., Charvat P. (2015). Review on Using Microencapsulated Phase Change Materials (PCM) in Building Applications. Energy Build..

[114] Hekimoğlu G., Sarı A., Kaygusuz K., Tyagi V.V., Sharma R.K., Gencel O. (2021). Thermal Management Performance and Mechanical Properties of a Novel Cementitious Composite Containing Fly Acid/Lauric Acid-Myristic Acid as Form-Stable Phase Change Material. Constr. Build. Mater..

[115] Sarı A., Hekimoğlu G., Kaygusuz K., Gencel O., Usta H., Sharma R.K. (2020). Evaluation of Pumice for Development of Low-Cost and Energy-Efficient Composite Phase Change Materials and Lab-Scale Thermoregulation Performances of Its Cementitious Plasters. Energy.

[116] Hekimoğlu G., Sarı A., Gencel O., Tyagi V.V. (2021). Silica Fume/Capric Acid-Stearic Acid PCM Included-Cementitious Composite for Thermal Controlling of Buildings: Thermal Energy Storage and Mechanical Properties. Energy.

[117] Cao V.D., Pilehvar S., Salas-Bringas C., Szczotok A.M., Rodriguez J.F., Carmona M., Al-Manasir N., Kjøniksen A.L. (2017). Microencapsulated Phase Change Materials for Enhancing the Thermal Performance of Portland Cement Concrete and Geopolymer Concrete for Passive Building Applications. Energy Convers. Manag..

[118] Dutkowski K., Kruzel M. (2021). Experimental Investigation of the Apparent Thermal Conductivity of Microencapsulated Phase-Change-Material Slurry at the Phase-Transition Temperature. Materials.

[119] Lemmon E.W., Huber M.L., McLinden M.O. (2010). Thermophysical Properties of Fluid Systems. NIST Chemistry WebBook.

[120] Zhang Y., Liu C., Qian F., Li T., Gao S., Wang Z., Kong X. (2020). Microencapsulated Phase Change Materials Composited Al_2_O_3_–SiO_2_ Aerogel and the Thermal Regulation Properties. J. Sol-Gel Sci. Technol..

[121] Long H., Liu F., Chen Z., Li Y., Ye S., Qin Y., Zhao J. (2024). Superhydrophobic Daytime Radiative Cooling Coating Incorporated with Phase Change Microcapsules for Building Thermal Regulation. J. Mater. Sci..

[122] Ali H.M., Arshad A., Janjua M.M., Sajjad U., Yan W.M. (2018). Thermal Management of Electronics: An Experimental Analysis of Triangular, Rectangular and Circular Pin-Fin Heat Sinks for Various PCMs. Int. J. Heat Mass Transf..

[123] Rostamian F., Etesami N., Mehrali M. (2024). Microencapsulation of Eutectic Phase Change Materials for Temperature Management of the Satellite Electronic Board. Appl. Therm. Eng..

[124] Jiang F., Qiu G., Wang X. (2022). Energy Harvesting and Thermal Management System in Aerospace. Front. Mater..

[125] Alshaer W.G., Nada S.A., Rady M.A., Baracu A., Ducu C., Minea A.A. (2015). Thermal Management of Electronic Devices Using Carbon Foam and PCM/Nanocomposite. Int. J. Therm. Sci..

[126] Zhu X., Zhang J., Zhu W., Yuan T., Fan X. (2020). Stable Microencapsulated Phase Change Materials with Ultrahigh Payload for Efficient Cooling of Mobile Electronic Devices. Energy Convers. Manag..

[127] Ren Q., Guo P., Zhu J. (2020). Thermal Management of Electronic Devices Using Pin-Fin Based Cascade Microencapsulated PCM/Expanded Graphite Composite. Int. J. Heat Mass Transf..

[128] Pause B. (2000). Textiles with Improved Thermal Capabilities through the Application of Phase Change Material (PCM) Microcapsules. Mell. Textilber. Int. Text. Rep..

[129] Hossain M.T., Khan T.M.Y., Chowdhury M.S.H., Sharmin N., Alam M.S. (2023). Fabrications, Classifications, and Environmental Impact of PCM-Incorporated Textiles: Current State and Future Outlook. ACS Omega.

[130] Salaün F., Devaux E., Bourbigot S., Rumeau P. (2010). Thermoregulating Response of Cotton Fabric Containing Microencapsulated Phase Change Materials. Thermochim. Acta.

[131] Shin Y., Yoo D.I., Son K. (2005). Development of Thermoregulating Textile Materials with Microencapsulated Phase Change Materials (PCM). II. Preparation and Application of PCM Microcapsules. J. Appl. Polym. Sci..

[132] Zhang W., Yi Z., Wang S. (2020). Preparation of PMMA/SiO_2_ PCM Microcapsules and its Thermal Regulation Performance on Denim Fabric. Pigment Resin Technol..

[133] Gu B., Li G., Zhang Q., Pan H., Duan M., Weng L., Zhao D. (2025). A novel method for increasing phase-change microcapsules in nanofiber textile through electrospinning. Adv. Funct. Mater..

[134] Xiao Q., Yuan W., Xu J., Li J., Gan G. (2019). Thermal Conductivity Enhancement of Hydrated Salt Phase Change Materials Employing Copper Foam as the Supporting Material. Sol. Energy Mater. Sol. Cells.

[135] Wang F., Yu J., Wang J., Xu S., Wu M. (2023). Progress in Application of Phase-Change Materials to Cooling Clothing. J. Energy Storage.

